# 

**Published:** 1814-06

**Authors:** 


### MONTHLY CATALOGUE OF MEDICAL BOOKS.

ADAMS'S Treatise on the supposed Hereditary Properties of Diseases, containing Remarks on unfounded Terrors and ill-judged Cautions consequent on such erroneous Opinions; with Notes illustrative of the Subject, particularly in Madness and Scrofula. 8vo. 5s. 6d.

Burnett's Practical Account of the Fever commonly called the Bilious Remittent, as it appeared in the Ships and Hospitals of the Mediterranean Fleet, with Cases and Dissections; to which are added, Facts and Observations illustrative of the Causes, Symptoms, and Treatment of Fever in the Mediterranean; comprehending the History of Fever in the Fleet, during the years 1810, 1811, 1812, 1813, and of the Gibraltar and Carthagena Fevers. 8vo. 10s. 6d.

Hey's Surgery. 8vo. 3d edition.

Hill's Essay on the Prevention and Cure of Insanity, with Observations on the Rules for the Detection of Pretenders to Madness. 8vo. 12s.

Pemberton on the Viscera. Third edition. 9s.

Reece's Chemical Guide, or complete Companion to the Portable Chest of Chemistry, being an Epitome of Modern Chemistry. 12mo. 7s. 6d.

---

### TO CORRESPONDENTS.

*In the next Number of the Journal, we shall have occasion to call our readers' attention to the new Bill for licensing houses for the reception of Insane Persons, about to be submitted to Parliament. A paper on this important subject, from Dr. Harrison, and one from a Physician with the signature X. Y. Z. have reached us, but quite too late for publication this month. We have also received communications from Messrs. Walker, Bernard, Rance, &c. &c. which will appear in due course.*

### INDEX

TO THE THIRTY-FIRST VOLUME.

ABEL, Mr. on hydrophobia 6
———— on Dr. Adams's suggesting depletion in hydrophobia 281
Abdominal viscera inflamed, fatal case of 372
Absorption, cutaneous, experiments upon 80
Abstinence, case of 50
Adams, Dr. Jos. his opinions on hydrophobia disputed 2, 281
———— on the appearance of the stomach after death 177
Adams, Mr. remarks on his recommendation of antim. tart. in ophthalmia 189
———— on his new operation for cataract 295
———— papers relating to his treatment of cataract 327
———— explanation respecting his name appearing in a newspaper 382
Ammonia, effects of, in preventing contagion 83
————, muriate of, efficacy of, in gangrene 518
ANALYSIS, CRITICAL, of recent publications 55, 143, 241, 321, 408, 489
Anasarca, case of, cured by digitalis in friction 432
Animal heat, experiments on 339
Antimonium tartarizatum, ineffectual in curing ophthalmia 17
————, its effects in ophthalmia considered 189, 355
Aorta, strong pulsation of, in the epigastric region 159
Apoplexy, case of, with the dissection 93
Apothecaries, remarks on the condition of 229
————, their report of diseases in London 171, 257, 346, 433, 522
————, London committee of, their third report 259
————, general meeting of 511
————, fourth report of the committee of 512
Armstrong, Dr. on caries of the spine 307
Arsenic, effects of, on the stomach 8
Babington, Dr. his case of the effects produced on olive oil in the stomach and intestines 45
————, case of fat forming in the intestines 46
Baillie, Dr. his case of hydrocephalus reviewed 68
————, ditto, with uncommon symptoms 69
————, on a strong pulsation of the aorta in the epigastric region 159
Barrow, Mr. his explanation respecting Mr. Adams 382
Baynton, Mr. his mode of treating diseases of the spine by rest 245
Belcombe, Dr. H. S. on acute rheumatism 269
Bladder, on the diseases of the 241
Blane, Sir G. bart. on the diseases of London 501
Blaquiere, Mr. G. on medical practice and diseases in Tripoli 478
Books in the press 173, 261
———— published at Leipzig, catalogue of 342
Bond, Mr. his case of dislocated spine 271
Borrett, Mr. on the resemblance between gastritis and hydrophobia 2
————, on the appearance of the stomach after death 3
Brain, disease of the, from external violence 506
Brande, Mr. result of his examination of the cæca of animals 47
Brodie, Mr. B. C. on the influence of the eighth pair of nerves on the stomach 486
Brooks, Mr. account of his museum 259
Burnett, Dr. W. on bilious remittent fever, reviewed 489
Cabanis, M. strictures on his practice in the case of Mirabeau 223
————, biographical memoir of 400

CASES—of abscess of the liver 9
———— of ophthalmia 18, 272
———— of vomiting urine 26, 106, 112, 362
———— of the effects of electricity 37
———— of delirium tremens 40
———— of concretion of olive oil and mucus in the intestines 45
———— of fat in the intestines 46
———— of extraordinary abstinence 50
———— of hydrocephalus 68, 69
———— of stricture and thickening of the ileum 71
———— of tetanus from wounds 73
———— of croup 81
———— of apoplexy 93
———— of diseased pancreas 95
———— of hydrophobia 122, 514
———— of convulsive affections 148, 373
———— of superfœtation 150
———— of mortification of the adipose membrane of the kidneys 156
———— of diseased liver 162, 353, 426, 447
———— of intestinal protrusion per anum 164
———— of tic douloureux 184, 380, 387
———— of luxation of the jaw 187
———— of effects of laudanum 193, 468
———— of fracture 213
———— of disease of the heart 217, 220
———— of diseased prostate, bladder, and rectum 225
———— of rabies in dogs 231
———— of diseased spine 246
———— of pemphigus 265
———— of acute rheumatism 269
———— of dislocated spine 271
———— of inflammation of the pericardium 274
———————— of the pleura 277
———— of diseased cervical vertebræ 308
———— of deafness 346, 379
———— of phrenitis 357
———— of fever 363
———— of abdominal inflammation 371
———— of partial perspiration 392
———— of impeded deglutition 393
———— of suppression of urine 398
———— of anasarca 432
———— of erysipelas 442
———— of wounds exposed to air 451
———— of fractured os humeri 456
———— of fractured cranium 485
———— of affection of the brain 497
CASES—of wound in the radial nerve 499
———— of diseased brain 506
———— of horns growing from a woman's head 518
Cataract, on Mr. Adams's treatment of, at Greenwich Hospital 326
————, practical treatise on, by Mr. Stevenson 336
Cavendish, Sir Thomas, account of a singular disease which affected his crew 306
Chevreuil, Mr. account of his experiment on the bitter principle of vegetable substances 519
Chincough, on the nature and treatment of 55
————, early symptoms of 56
————, on the fever accompanying 60
————, appearances on dissection 61
Chlorine, account of 338
Christ's Hospital, report of the diseases at 479
Cleveland, Mr. on the medical effects of electricity 37
Climacteric disease, account of the 163
Clothing, importance of attending to, in pulmonary diseases 371
COLLECTANEA MEDICA 50, 136, 235, 304, 392, 478
Combe, Dr. his fatal case of stricture of the ileum 72
Cornish, Mr. on the fatal effects of laudanum 193
————, on a convulsive epidemic in Cornwall 373
————, strictures on his account of ditto 464
Crane, General, case of 451
Crichton, Dr. on vitality 431
Croup, instance of recovery from 81
————, observations upon 358
————, remedy for 518
Davy, Sir Humphry, his account of iode 256
————————, his account of fluorine 337
Davy, Mr. John, on animal heat 339
Deafness cured by puncturing the tympanum 346
———————— by a simple process 379
Deaths within the bills of mortality, for 1813 84, 258, 347, 434, 522
Deglutition, impeded, observations on 392
————, case of 393
Delirium tremens, case of 40
Denmark, Mr. his case of wound of the radial nerve 499
Diabetes mellitus, facts respecting 153
Digitalis, application of friction efficacious in anasarca 432

Diseases in London, monthly average of 171, 257, 346, 433, 522
——— quarterly report of, at Christ's Hospital 479
——— comparison of, in public and private practice 501
M'Donald on a convulsive epidemic in Cornwall 464
Drugs, prices of 87, 174, 262, 348, 435, 523
Elastic gum catheter, useful after lithotomy 170
Elaterium, beneficial effects of, in dropsy 144
Electricity, medical effects of 37
Emetic, good effects of, in ophthalmia 272
Epiglottis, use of, in deglutition 318, 463
Erysipelas, a contagious disease 441
———, cases of 442
Eyes, peculiar affection of the, described by Dr. Heberden 147
———, inflammation relieved by evacuating the aqueous humour 504
Fanaticism, remarkable effects of, in Cornwall 373
Fat, on the formation of, in the intestines of living animals 43
———, case of, forming in the intestines 46
Ferrier, Dr. his Medical Histories and Reflections, vol. iv. 143
Fever, contagious, at Gibraltar, account of 258
———, cases of, relieved by the application of leeches 364
———, bilious remittents in the Mediterranean 489
Field, Mr. on the diseases at Christ's Hospital 479
Flesh, human, instances of its being converted into adipocire or spermaceti 45
Fluorine, account of 337
Fooks, Ann, examination and vindication of, by the Rev. J. Grimshaw 19
———, account of her case by Dr. Yeats 26
———, observations on her case, by Mr. Gibbon 25, 205
———, ditto, by Mr. Pulley 196
Foot, Mr. Jessé, his case of diseased prostate, bladder, and rectum 225
Fossil bones at Guadaloupe, account of 339
——— human skeleton 432
Fothergill, Dr. J. remarks upon his recommendation of hemlock in tic douloureux 383
Frogley, Mr. his case of pemphigus 265
Furor uterinus, remarks upon 213
Gallois, Dr. Le, on the vital principle 417
Gangrene remedied by muriate of ammonia sprinkled on the affected part 518
Geoghegan, Mr. his commentaries on the venereal disease reviewed 408, 495
Gibbon, Mr. his animadversions on Dr. Yeats 22
———, observations on Ann Fooks's case 125, 205
Ginguené, his biographical account of M. Cabanis 400
Girdlestone, Dr. on urinous vomiting 112
———, on the Mal d'Aleppo 282, 372
Gout, theory of 13
Grimshaw, Rev. J. S. his vindication of Ann Fooks 19
———, remarks upon his interference in Ann Fooks's case 196
Halford, Sir Henry, on the climacteric disease 163
Hannah, appearance of his stomach on dissection after execution 4
Heart, some diseases of, described 217
———, inflammation of, caused by passions and intemperance 220
Heberden, Dr. on a peculiar affection of the eyes 147
———, his reasons for the superior healthiness of London 150
Heineke, Dr. on impeded deglutition 393
Hemlock, effects of, in tic douloureux 383
Home, Sir Everard, on the formation of fat in the intestines 43
———, account of his Hunterian oration 344
Horns, case of, growing from the head of a young woman 518
Hosack, Dr. on exposing wounds to air after operations 450
———, on pneumonia typhodes 471
Hunter, Mr. John, remarks upon some of his opinions 178
Hutchinson, Dr. his case of diseased brain 506
Hydrocephalus, cases of, in which the bones of the skull were separated 69
Hydrophobia, remarks upon 4-6
———, appearances of, on dissection 166
———, case of 122, 514
Jackson, Dr. James, on tic douloureux 179, 383
James's powder, efficacy of, in tetanus 74
Ileum, stricture of 71

Indians (North American) account of their mode of curing diseases 209
Insects, anatomy of, observations on the 515
Integuments, human, observations on, by Dr. Ramsay 458
INTELLIGENCE, medical and philosophical 80, 167, 255, 337, 431, 511
Iode (a new gas) account of 255
Jones, Mr. W. his case of abdominal inflammation 371
Ipecacuanha, pulv. comp. efficacy of, in tetanus 75
Italy, state of medicine in, in 1812 81
Kington, Mr. his case of luxation of the jaw 188
Kœnig, Mr. C. on fossil bones 339
Lane, John, his account of a singular disease which affected the men of Sir T. Cavendish's ship 306
Laplanders, diseases of 51
Latham, Dr. on tetanus 73
———, on tumours mistaken for diseases of the liver 78
———, on symptoms mistaken for those of angina pectoris 161
Laudanum, fatal effects of 193
———, instance of recovery from 468
LECTURES announced—Theory and Practice of Medicine—Dr. Powell 85
——— Dr. Hooper and Dr. Ager 173, 521
——— Dr. Adams 173, 521
——— Dr. Watt 346
——— Dr. Clutterbuck 521
——— Dr. Harrison (Clinical cases) 521
——— Anatomy, Surgery, and Physiology—Mr. Abernethy 85, 170
——— Mr. Taunton 85, 433
——— Mr. Brooks 85, 521
——— Mr. Carpue 433
——— Midwifery, and Diseases of Women and Children—Dr. Squire 86, 261, 521
——— Dr. Clough 86
——— Dr. and Mr. Clarke 86
——— Dr. Merriman 86
——— Dr. Gooch 173
——— Dr. Davis 433, 522
——— Mr. Hopkins 433
——— Chemistry and Materia Medica—Dr. Hill 85
——— Mr. Singer 86
——— Dr. Clutterbuck 521
——— Dr. Hooper, and Dr. Ager 173, 521
——— Dr. Prout 173
——— On the Teeth—Mr. Fuller 173
——— On the Eye and Ear—Mr. Stevenson 86, 256
Lee, Mr. on the effects of ammonia in preventing contagion 83
Leeches, application of, beneficial in fever 364
Leipzig fair, catalogue of books published at 342
Liver, abscess of 9
———, diseased 353, 447
———, on the diseases of 425
London, diseases in, monthly average of 171, 257, 346, 433, 522
———, healthiness of, accounted for 150
Lucas, Mr. on acute rheumatism 89
Lungs, on the black spots of, by Dr. Pearson 128
Mackesy, Mr. his case of fracture 214
Magendie, M. on the use of the epiglottis in deglutition 318
Mal d'Aleppo, description of the 282
———, remarks on, by Dr. Girdlestone 372
Marshall, Mr. S. his case of suppression of urine, succeeded by gangrene 398
Maton, Dr. on superfœtation 151
Measles, fatality of, supposed to be influenced by vaccination 66
———, arguments against their being affected by vaccination 273
Medical Transactions, by the College of Physicians, vol. iv. 67
——— reformists, on the motives of 91
——— apprenticeships, abuses of 99
——— histories and reflections, by Dr. Ferriar 143
——— economy, observations upon 321
———, review of, considered 415
——— practice, plan for reforming 324
———, among certain tribes of Indians 304
———, in Tripoli 478
Medicine, state of, in Italy 81
———, in Spain 235
Medico-chirurgical Transactions, critical analysis of 496
Meglin, Mr. on tic douloureux 380
Mercurial action, efficacy of, in diseased liver 447
———, in affections of the brain 497
Meteorological register 88, 175, 263, 351, 439, 527
Midwifery, practice of, among the Toccoupinamboultii 305
Mirabeau, M. detail of his last illness, and dissection 221, 224
Mollities ossium, analysis of the bones in 498
Monthly catalogue of medical books 88, 176, 264, 352, 440, 528

Morbid anatomy, collections of 274
Morgan, C. his case of deafness 379
Murat, John, his successful introduction of vaccination on the banks of the Tygris 520
Murphy, Mr. remarks on his history of the human teeth 302
Muscles, on the vis insita of 288
Nauche, Dr. on diseases of the bladder 241
Nerves, influence of the eighth pair on the stomach 487
Nipples, chapped, remedy for 258
Nitrate of silver, use of, in convulsive affections 148
Notice to correspondents 88, 176, 264, 440, 528
Ophthalmia, inefficacy of antim. tart. in the cure of 17
——, case of 18
——, cured by an emetic 272
——, remarks upon 189, 355
Oration, Hunterian, account of the 344
Os humeri, case of compound fracture of 456
Pancreas, diseases of the 94
Patagons, practice of medicine among the 304
Pearson, Dr. George, on the colouring matter of the black bronchial glands 128
Pelletan, the Chevalier, on diseases of the heart 217
Pemphigus, case of 265
Penis, arteries of the, delineated 211
——, erysipelatous, inflammation of the 412
Pericardium, inflammation of the 274
Perry, Mr. on delirium tremens 40
Perspiration, partial, case of 392
Petersburgh, temperature of 54
Phrenitis, case of, consequent on injury of the head 357
Phymosis, observations upon 410
——, treatment of 413
Pleura, inflammation of the 277
Pneumonia typhodes, remarks upon 471
Poppy heads, white, smoke of, equally efficacious, and less deleterious, than that of stramonium 171
Portal, Dr. Antoine, on diseases of the liver 425
Powell, Dr. on the use of nitrate of silver in convulsive affections 148
Priapism, observations on, by Dr. Ramsay 211
Prize, jacksonian, adjudged to Mr. Pring 521
—— questions proposed 517
Prostate, enlarged, case of 226
Pulley, Mr. on the case of Ann Fooks 196
Pulmonary consumption, on the treatment of 298, 365
Rabies canina, cases of 231
Rain, annual quantity of, in Sweden 54
Ramsay, Dr. on the causes of priapism 211
——, account of his treatise on anatomy 249
——, on the vis insita musculorum 288
——, on the human integuments 458
Raucedo catarrhalis, sometimes mistaken for croup 358
Regimen, importance of, in the cure of diseases 370
Rheumatism, acute, on the cure of 89
——, cured by cold applications 269
Rigby, Mr. his report on vaccination 136
——, on the improbability of measles being influenced by vaccination 273
Satterley, Dr. on hydrophobia 166
Semple, Mr. on abscess of the liver 9
——, Mr. R. his case of diseased liver 447
Senter, Dr. objections to his case of urinous vomiting 203
Sewall, Dr. on diseases of the pancreas 94
Shearly, Mr. his case of fractured cranium, with laceration of the dura mater 485
Silica, account of 337
Society, royal, proceedings of 167, 255, 337, 431
——, medical, of London, anniversary of 256
——, Linnæan 340
—— for preventing accidents in coal-mines 341
——, new rupture, account of 163
—— of pharmacy, of Paris 517
Spain, state of medicine in 235
Spider, effects of, in the ear, remedied by oil 172
Spine, diseases of, successfully treated by rest 245
——, diseased, case of 246
——, distension of, &c. 271
——, treatment of 313
Stevenson, Mr. account of his introductory lecture 256
——, on cataract 336
Stockholm, table of the temperature of each month in the year 54
Stomach, appearance of, after sudden death 4, 178
——, ditto, from the effects of arsenic 8

Superfœtation, case of 151
Surgeons, royal college of, lectures delivered at the 170
Sweden, annual quantity of rain in 54
Temperature of Stockholm and Petersburgh 54
Tetanus from wounds, good effects of sudorifics in 73
Tic douloureux, history of 180
——, symptoms of 181
——, cases of 184, 380, 387
——, observations upon 383
Tobacco, broad-leaved, analysis of 519
Toccoupinambaultii, their practice of midwifery and medicine 305
Transactions of societies 167, 255, 337, 431, 340
Tullidge, Mr. his case of diseased liver 353
Turner, Dr. his case of mortification of the adipose membrane of the kidneys 157
——, description of the appearances on dissection 158
Vaccination, influence of, on measles 66
——, Norwich report on 136
——, account of its introduction on the banks of the Euphrates 520
Vaccine matter, ingenious mode of preserving 432
Vauquelin, M. his analysis of broad-leaved tobacco 519
Venereal disease, Mr. Geoghegan's treatise upon, reviewed 409
——, aggravated by the wretched condition of the military hospital at Toulon 412
——, cured in Portugal without mercury 497
Vertebræ, cervical, case of diseased, terminating by anchylosis 308
Vipers, venom of, remedied by ammoniac 172
Vital principle, observations upon the, by Dr. Le Gallois 417
Vitality, constant waste of, supplied by food 431
Vomiting and nausea, effects of, in ophthalmia 17
——, full, ineffectual in counteracting the effects of a large dose of laudanum 193
Urine, suppression of, succeeded by gangrene of the arm 398
Urinous vomiting, cases of 26, 106, 112, 362
——, possibility of, supported 359
Uva ursi, inefficacy of, in pulmonary consumption 299
Walker, Mr. on pulmonary consumption 296
Want, Mr. on the use of the epiglottis 463
Wardrop, Mr. on evacuating the aqueous humour 504
Warren, Dr. P. on diabetes mellitus 153
——, Dr. J. C. on inflammation of the pericardium 274
——, on inflammation of the pleura 277
Watt, Dr. on chincough 55
Wayte, Mr. his case of fractured os humeri 456
Weatherhead, Mr. on erysipelas 441
Wounds, advantage of exposing them to air after operation 450
Yeatman, Mr. his case of recovery from laudanum 468
Yeats, Dr. account of Ann Fooks's case 26, 106
——, strictures upon his conduct and opinions in the case of Ann Fooks 196, 205
Youatt, Mr. on rabies canina, with cases 231
Young, Dr. remonstrance to the editors of the Edin. Med. and Surg. Journal 12

